# Supplementary material for: Content-rich biological network constructed by mining PubMed abstracts
Source: BMC Bioinformatics. 2004 Oct 8;5:147. doi: 10.1186/1471-2105-5-147 (PMC528731; doi:10.1186/1471-2105-5-147)
Supplement: Additional File 2 — The original results of the above study (non-essential files are deleted to keep the file size under the limit set by BMC bioinformatics). [file 1471-2105-5-147-S2.bz2 › chilibotAdditionalFile2/dip05/562ID11502794E10923/html/IGF2_IGF2R.html]

 


 **IGF2** and **IGF2R** 
  
Found 192 abstracts in PubMed, retrieved 05.  
 

 What does Google say? 
 PDF only 
| .edu only 

---

**Interactive relationship** (e.g. stimulation, inhibition, etc)

**Non-interactive relationship** (e.g. studied together, co-existance, homology, etc.)

- Familial aggregation of abnormal methylation of parental alleles at the  **IGF2**  H19 and  **IGF2R**  differentially methylated regions.  Ref: 12812984 Hum Mol Genet< MedlineTA>Hum Mol Genet,
- We examined allelic methylation ratios at DMRs within the  **IGF2**  H19 and  **IGF2R**  loci in a panel of 48 three generation families.  Ref: 12812984 Hum Mol Genet< MedlineTA>Hum Mol Genet,
- Moreover, analysis of imprinted gene expression in a methyltransferase knockout Dnmt1 mouse has shown that the imprint is lost in a number of cases, resulting in either two silent alleles  **Igf2** ,  **Igf2r**  and Kvlqt or two expressed alleles H19, p57kip2, Snrpn, and Xist 0 12.  Ref: 12843456 Methods Mol Biol, 2001
- The  **IGF2**  receptor  **IGF2R**  is a USF2 specific target in nontumorigenic mammary epithelial cells, but not in breast cancer cells.  Ref: 12857727 J Biol ChemJ Biol Chem, 2001
- In addition, we have extended physical maps of sheep and goat as 11 genes HSD3B1, INHBA, CSN10,  **IGF2R** , PIGR, MAP1B, DSC1, ELN, TNFRSF6, CGN1,  **IGF2**  and 14 genes SOD1, HSD3B1, CSN10,  **IGF2R** , RB1, TG, PIGR, MAP1B, IGH@, LTF, DSC1, TNFRSF6, CGN1,  **IGF2**  were assigned for the first time to goat and sheep chromosomes, respectively.  Ref: 12873218 Anim Genet, 2003
